# Animacy enhances recollection but not familiarity: Convergent evidence from the remember-know-guess paradigm and the process-dissociation procedure

**DOI:** 10.3758/s13421-022-01339-6

**Published:** 2022-06-21

**Authors:** Gesa Fee Komar, Laura Mieth, Axel Buchner, Raoul Bell

**Affiliations:** Department of Experimental Psychology, Heinrich Heine University Düsseldorf, 40225 Düsseldorf, Germany

**Keywords:** Animacy advantage, Recollection, Familiarity, Remember-know-guess paradigm, Process-dissociation procedure

## Abstract

Words representing living beings are better remembered than words representing nonliving objects, a robust finding called the animacy effect. Considering the postulated evolutionary-adaptive significance of this effect, the animate words’ memory advantage should not only affect the quantity but also the quality of remembering. To test this assumption, we compared the quality of recognition memory between animate and inanimate words. The remember-know-guess paradigm (Experiment 1) and the process-dissociation procedure (Experiment 2) were used to assess both subjective and objective aspects of remembering. Based on proximate accounts of the animacy effect that focus on elaborative encoding and attention, animacy is expected to selectively enhance detailed recollection but not the acontextual feeling of familiarity. Multinomial processing-tree models were applied to disentangle recollection, familiarity, and different types of guessing processes. Results obtained from the remember-know-guess paradigm and the process-dissociation procedure convergently show that animacy selectively enhances recollection but does not affect familiarity. In both experiments, guessing processes were unaffected by the words’ animacy status. Animacy thus not only enhances the quantity but also affects the quality of remembering: The effect is primarily driven by recollection. The results support the richness-of-encoding account and the attentional account of the animacy effect on memory.

## Introduction

An evolutionary-adaptive, function-based account of human cognition (Nairne & Pandeirada, 2016) predicts that animate beings are prioritized in cognitive processing relative to inanimate objects (Nairne et al., 2013; Nairne et al., 2017). The distinction between animate beings and inanimate objects is fundamental for perception and attention (New et al., 2007), occurs early in development (Opfer & Gelman, 2011), and is present at the neurorepresentational level (Caramazza & Shelton, 1998). Extending this line of research, animacy has been established as an important determinant of memory (Nairne et al., 2017). Specifically, it has been reported that words referring to animate beings (henceforth animate words) are better remembered than words referring to inanimate objects (henceforth inanimate words). This finding has become known as the *animacy effect* on memory (Nairne et al., 2013). While the animacy effect has robustly been found in free-recall paradigms (Bonin et al., 2014; Leding, 2019; Meinhardt et al., 2018, 2020; Popp & Serra, 2016, 2018), it is less clear whether there is a robust animacy advantage in recognition paradigms. Some researchers have reported enhanced recognition of animate in comparison to inanimate words (Bonin et al., 2014), but inconsistent results have also been obtained (Leding, 2020; Mieth et al., 2019). For instance, Leding (2020) reported that animacy induced a guessing bias towards believing the words had occurred before, but the animacy status did not affect memory accuracy. A potential reason for these inconsistent findings is that animacy might not equally enhance all processes underlying observable recognition memory performance. Theories with a focus on elaborative encoding (Meinhardt et al., 2020) and attention (Bugaiska et al., 2019) allow predicting animacy to specifically enhance recollection but not familiarity. The present study serves to test this hypothesis by examining both subjective and objective aspects of remembering using the remember-know-guess paradigm (Gardiner et al., 1996; Gardiner et al., 1997) and the process-dissociation procedure (Jacoby, 1991), respectively. Furthermore, we will adopt a multinomial modeling approach to capture process-pure measures of recollection and familiarity and to clearly distinguish between memory and guessing processes.

Since the animacy effect on memory was first introduced (Nairne et al., 2013), enhanced free recall of animate as compared to inanimate words has been demonstrated with different types of intentional (Bonin et al., 2015; Félix et al., 2019; Nairne et al., 2013) and incidental encoding conditions, such as pleasantness-rating or animacy-categorization tasks (Bonin et al., 2014; Félix et al., 2019; Gelin et al., 2017). An animacy advantage has been found with words in different languages (in French, Bonin et al., 2014; in Portuguese, Félix et al., 2019; in German, Meinhardt et al., 2018; in English, Nairne et al., 2013), pictures (Bonin et al., 2014), and pseudowords that were associated with properties characteristic of animate beings or inanimate objects (VanArsdall et al., 2013). Although there seem to be some boundary conditions (Kazanas et al., 2020; Popp & Serra, 2016), the effect has been demonstrated to generalize to different types of memory tests, such as paired-associate recall (VanArsdall et al., 2015), serial-order recall (Daley et al., 2020), and source-memory tests (Gelin et al., 2018; Mieth et al., 2019). Only recently, a large-scale study on word memorability (Madan, 2021) confirmed the original finding of Nairne et al. (2013) that a word’s animacy status explains more variance in free recall than other dimensions, such as concreteness or age of acquisition, and thus may be one of the most relevant semantic word dimensions for predicting memory performance.

Based on the adaptive-memory framework proposed by Nairne and co-workers (for a review, see Nairne & Pandeirada, 2016), it has been postulated that cognitive systems have been evolutionarily tuned to solve adaptive problems. Prioritizing information about animate beings over information about inanimate objects might have increased the inclusive fitness of our ancestors because animate beings, such as predators, prey, and mating partners, entail a high significance for achieving specific adaptive goals related to survival and reproduction (Nairne & Pandeirada, 2008). Regardless of the animacy effect’s potential evolutionary-adaptive value, however, there is still much to learn about the immediate proximate mechanisms underpinning its expression (for how to distinguish ultimate and proximate explanations in evolutionary theory, see Scott-Phillips et al., 2011). Several such mechanisms have already been ruled out as they failed to gain empirical support, including categorical recall strategies (Nairne et al., 2013; Serra, 2021; VanArsdall et al., 2017), emotional arousal (Meinhardt et al., 2018; Popp & Serra, 2018), and mental imagery (Blunt & VanArsdall, 2021; Gelin et al., 2019).

By contrast, both the *richness-of-encoding account* and the *attentional account* have received some empirical support and thus offer promising explanations of the animacy effect. Following a proposition by Nairne et al. (2017), Meinhardt et al. (2020) found that animate words spontaneously stimulated participants to generate more ideas than inanimate words. Already established as a proximate mechanism underlying the survival-processing effect (Erdfelder & Kroneisen, 2014; Kroneisen et al., 2013; Kroneisen et al., 2014, 2016; Kroneisen & Erdfelder, 2011; Röer et al., 2013), the richness-of-encoding account may thus also provide a proximate explanation of the animacy effect. Specifically, animate items may benefit from a richer encoding context that may provide more distinctive memory traces and thus a larger set of retrieval cues at test (see also Bonin et al., 2022). According to the attentional account (Bugaiska et al., 2019), animate items are more likely to draw attention during encoding than inanimate items. Bonin et al. (2015) did not find evidence for the animacy effect to decrease under cognitive load but reported in one of three experiments that secondary task performance suffered when target words denoted animate items, consistent with the idea that animate items capture attention. In line with these results, Bugaiska et al. (2019) showed that participants were slower to name the font color of animate words than that of inanimate words in the Stroop paradigm, further supporting the idea that attentional resources are drawn to the animate words. Leding (2019) required her participants to perform the encoding task and the secondary task simultaneously. Unlike Bonin et al. (2015), who administered the primary and secondary task consecutively, she found a reduced animacy effect on free recall under divided attention compared to the full-attention condition, suggesting that at least part of the processing advantage of animate words is attributable to an increased allocation of attentional resources to the animate words. This finding is at odds with that from a study by Rawlinson and Kelley (2021), who also used a simultaneous dual-task paradigm but found that the word-type-by-attention interaction was not significant in both free recall and recognition. However, Rawlinson and Kelley provided evidence suggesting that animate beings are more richly represented than inanimate objects.

While the results are still rather mixed, the richness-of-encoding and the attentional account converge in that animacy should affect not only the quantity of remembering but also its quality. For instance, the richness-of-encoding account implies that animate compared to inanimate items elicit more ideas during encoding that later serve as retrieval cues at test. This implies that participants should remember not only the items themselves but also the cognitions that were active while encoding the item. In recognition tests, memory for previously encountered animate items should therefore be enhanced not only by feelings of familiarity but also by the recollection of associated thoughts. Similarly, increased attentional prioritization during encoding should selectively enhance recollection but not familiarity (Gardiner & Parkin, 1990).

To understand potential moderators of the animacy effect, it thus seems promising to examine how animacy affects the quality of recognition. According to dual-process theories (for a review, see Yonelinas, 2002), recognition memory is composed of a controlled retrieval process referred to as *recollection* and an automatic process yielding an item’s *familiarity*. Only in the memory state of recollection are participants able to recall specific contextual details of the encoding episode (e.g., the thoughts that came to mind while reading a word). Familiarity-based recognition solely involves an acontextual feeling that an item occurred before. Recollection is assumed to require attention during encoding and retrieval, while familiarity may be less likely to rely on such controlled processing (Gardiner et al., 1996; Gardiner & Parkin, 1990; Yonelinas, 2002).

To date, studies examining animacy effects on recognition are scarce and rely predominantly on the *remember-know paradigm* introduced by Tulving (1985) and further developed by Gardiner (1988). The remember-know paradigm is considered as an extension of the classical old-new recognition paradigm and requires participants to qualify their experiential retrieval states following an “old” judgment as “remembered” or “known.” “Remember” judgments are reserved for those memories that involve rich recollections of the encoding episodes. “Know” judgments, by contrast, are to be given when participants experience only feelings of familiarity but do not have rich and vivid experiences of remembering. In the *remember-know-guess paradigm*, participants are also allowed to indicate that they classified a word as “old” based on guessing (Gardiner et al., 1996; Gardiner et al., 1997).

Regarding the animacy effect, Bonin et al. (2014) found a higher number of “remember” judgments for recognized animate than inanimate words, while neither the number of “know” judgments nor the number of “guess” judgments differed between animate and inanimate words. These findings were replicated in two additional studies (Bugaiska et al., 2016; Rawlinson & Kelley, 2021) in which animacy selectively enhanced “remember” judgments without affecting “know” judgments, while false-alarm rates were unaffected. However, Leding (2020) found an increase in false-alarm rates for animate words. Beyond that, the animacy effect on recognition was eliminated once response bias was taken into account, suggesting that animacy primarily affected guessing but not recognition accuracy. Stimulated by these inconsistencies, the present study serves to reassess the effect of animacy on the quality of experiential retrieval states with the remember-know-guess paradigm involving a new set of words. Remember-know paradigms, however, are not free from criticism. For instance, emotional events are often associated with an intensified subjective experience of recollection compared to neutral events even if the accuracy of the objective memory for contextual details is not enhanced (Sharot et al., 2004; Talarico & Rubin, 2003). The enhanced rate of “remember” judgments for emotional words may thus reflect a more intense emotional experience without affecting memory accuracy. Analogously, it is possible that animate words are more likely to be judged as being “remembered” only because they elicit subjective feelings of vividness which, however, may be unrelated to the mnemonic status of the words. Simply providing a “guess” category is unlikely to solve the problem that animate words may be experienced as more vivid at test.

The present study offers two remedies to these issues. First, in Experiment 1, we reapply the remember-know-guess paradigm to establish a link to prior research (e.g., Bonin et al., 2014) but use the well-validated multinomial *four-states model of memory retrieval experiences* (Erdfelder et al., 2007) to separately measure recollection-based memory processes (experiencing memory of an item including the circumstances in which the item has been encountered), familiarity-based memory processes (experiencing memory of an item without detailed contextual integration), guessing (selecting an item in the absence of memory), and the detection of new words (as not having been encountered before). The model yields parameters that reflect recollection and familiarity without being contaminated by guessing. Recollection and familiarity can thus be measured and compared at the level of the postulated latent memory processes underlying the words’ observable classification in the memory test. A major advantage of the four-states model is that it has been empirically validated: The model’s recollection parameter has been shown to be sensitive to depth-of-encoding manipulations while remaining unaffected by response-bias manipulations that only affect the model’s guessing parameters (Erdfelder et al., 2007). A second extension of prior studies is that we do not rely on the remember-know-guess paradigm alone but complement this approach using the process-dissociation procedure (Jacoby, 1991) in Experiment 2 to objectively distinguish between contributions of recollection, familiarity, and guessing processes to performance in recognition memory tasks. Specifically, we use the well-validated *multinomial process-dissociation model* (Buchner et al., 1995) to separately measure memory and guessing processes. By examining animacy effects on both subjectively experienced retrieval states (Experiment 1) and objective memory performance (Experiment 2), we aim to test whether these two approaches provide convergent evidence on the effect of animacy on recognition. Both the richness-of-encoding account (Meinhardt et al., 2020) and the attentional account (Bugaiska et al., 2019) allow us to derive the prediction that the animacy effect should be primarily driven by the enhanced recollection of animate compared to inanimate words. These accounts thus lead to the hypothesis that animacy should selectively enhance recollection but not familiarity.

## Experiment 1

### Method

#### Participants

The online experiment was implemented using SoSci Survey (Leiner, 2020) and made available through https://www.soscisurvey.de. Participation was only possible with a desktop or laptop computer, not with a smartphone or tablet. The experiment was advertised on social media and via email. All participants were students. The final sample consisted of 110 participants (99 female) with a mean age of 22 years (*SD* = 5). Twenty-nine additional data sets could not be included in the analysis: 26 participants did not complete the experiment (ten participants dropped out even before they had given informed consent, presumably following our instructions to close the browser window if they were not in a distraction-free environment; six dropped out when they were asked about their demographic data; ten dropped out after they had started the encoding task), and three participants were under 18 years old and thus not of legal age in Germany (which is a requirement for consenting on the use of their data). We aimed for a sample size of at least 100 valid data sets and stopped data collection at the end of the day on which this criterion was reached. A sensitivity analysis with G*Power (Faul et al., 2007) revealed that, with a sample size of *N* = 110, 160 responses in the recognition test, and α = .05, an effect of animacy on the four-states model’s recollection and familiarity parameters of the size *w* = 0.03 could be detected with a statistical power of 1 – β = .95. Participation was compensated by course credit or the chance to win a € 20 voucher for a popular online store. All participants gave written informed consent prior to participation. Approval was obtained from the ethics committee of the Faculty of Mathematics and Natural Sciences at Heinrich Heine University Düsseldorf for a series of animacy experiments to which the present experiment belongs. However, minor adjustments were necessary due to the COVID-19 pandemic that did not require additional ethics approval. The experiment was conducted in accordance with the Declaration of Helsinki.

#### Materials

Following the procedure of Meinhardt et al. (2020), we obtained the word materials from the database of German words by Schröder et al. (2012). Lists of animate and inanimate words (80 words each) were matched on ten mnemonically relevant dimensions (see Table 1). The norming data were taken from the norming studies by Schröder et al. (2012) and Meinhardt et al. (2020).

**Table 1:** Dimensions on which animate and inanimate word lists were matched in Experiment 1

Dimension (range of the rating scale)	Animate		Inanimate		Comparison
	*M*	*SD*	*M*	*SD*	
Semantic typicality (1–7)	2.37	0.94	2.58	1.36	*t*(140.12) = 1.11, *p* = .267, *d* = 0.18
Age of acquisition (1–7)	3.35	1.21	3.40	1.18	*t*(158) = 0.25, *p* = .806, *d* = 0.04
Conceptual familiarity (1–5)	3.19	0.57	3.34	0.83	*t*(140.65) = 1.34, *p* = .183, *d* = 0.21
Word frequency	9.54	17.65	9.00	18.27	*t*(158) = 0.19, *p* = .850, *d* = 0.03
Number of phonemes	5.05	1.57	4.90	1.36	*t*(158) = 0.65, *p* = .518, *d* = 0.10
Number of syllables	2.03	0.81	1.99	0.67	*t*(152.21) = 0.32, *p* = .750, *d* = 0.05
Number of letters	6.15	1.66	5.74	1.52	*t*(158) = 1.64, *p* = .104, *d* = 0.26
Concreteness (1–7)	5.17	0.51	5.08	0.54	*t*(158) = 1.09, *p* = .276, *d* = 0.17
Meaningfulness (1–7)	3.66	0.72	3.60	0.75	*t*(158) = 0.51, *p* = .614, *d* = 0.08
Imagery (1–7)	5.47	0.96	5.46	1.00	*t*(158) = 0.11, *p* = .912, *d* = 0.02

Norms provided by Schröder et al. (2012) were taken to determine semantic typicality, age of acquisition, conceptual familiarity, word frequency (based on dlexDB database, Heister et al., 2011), number of phonemes, and number of syllables of the animate and inanimate words. Values for the number of letters, concreteness, meaningfulness, and imagery were taken from the norming study by Meinhardt et al. (2020). Words were selected so as to minimize, for each dimension, the mean differences between the lists of animate and inanimate words. The rightmost column shows that the lists of animate and inanimate words did not differ significantly on any of the controlled dimensions

#### Procedure

In the incidental encoding phase, a mixed list of 40 animate and 40 inanimate words was presented in random order. These words were randomly selected from the set of 80 animate and 80 inanimate words for each participant; the remaining 80 words were used as new words in the unexpected recognition test (see below). The present experiment was modeled after a study by Bonin et al. (2014) in which an animacy-categorization task was used. In the present experiment, participants had to rate the animacy status of each word on a seven-point scale ranging from *certainly inanimate* (1) to *certainly animate* (7). Each word was displayed in 20-point Arial font at the center of the browser window until participants initiated the presentation of the next word. Recording of the response times at encoding began when the word was presented and ended when the participant clicked on the “next” button.

The recognition test was separated from the encoding phase by a short distractor task lasting 44 s (*SD* = 14) on average, in which participants completed ten simple mathematical equations such as “15 – 7 = ?” (*M* = 98 % correct). In the recognition test, the 80 old words from the encoding phase were randomly intermixed with 80 new words (40 animate, 40 inanimate), resulting in 160 words being presented. The participants were instructed to indicate for each word whether it was “old” or “new.” Conditional upon a word being judged as “old,” participants were also asked to indicate the quality of their memory according to the remember-know-guess paradigm (Gardiner et al., 1996; Gardiner et al., 1997). The instructions read:

For some words, you will recollect exact details of the circumstances in which you saw the word. For example, you may precisely recollect the type face or the thoughts that crossed your mind while reading the word. Then you have a “detailed recollection” of the word. For other words, however, you will have a “feeling of familiarity.” You then only know that the word is old without recollecting details of the circumstances in which you learned the word. Still other words you will judge as “old” based on “guessing.” You then have neither a detailed recollection nor a feeling of familiarity but merely guess that the word may have been present in the first phase.

For each word classified as “old,” participants indicated whether their classification was based on a “detailed recollection,” a “feeling of familiarity,” or “guessing.” These labels were chosen because the present experiment served to measure recollection and familiarity. In fact, participants do not always seem to intuitively understand the labels of the remember-know-guess paradigm in its canonical form (Umanath & Coane, 2020). In German, the language in which the experiment was conducted, the literal translation of “I know” conveys a particularly strong confidence in one’s memory, while “I remember” indicates a relatively lower level of confidence in one’s memory. However, the labels “detailed recollection” and “feeling of familiarity” reflect the to-be-measured constructs less ambiguously and were thus used, together with detailed instructions. The “guessing” response category was included because it has been found to improve the precision of parameter estimation in the four-states model (Erdfelder et al., 2007). Participants clicked the “next” button to proceed.

After the recognition test, participants were asked to report whether they had followed the instructions and whether all stimuli had been accurately presented.^1^ Thereafter, participants were compensated, debriefed, and thanked for their time. The experiment took about 30 min.

### Results

#### Ratings and response times

As expected, animacy ratings were higher for animate words (*M* = 6.45, *SE* = 0.07) than for inanimate words (*M* = 1.97, *SE* = 0.11), *t*(109) = 26.39, *p* < .001, *d*_z_ = 2.52. After applying an outlier correction (excluding response times that deviated by ± 3 *SD* from the individual mean), it was found that participants rated animate words (*M* = 3,442 ms, *SE* = 106 ms) faster than inanimate words (*M* = 3,738 ms, *SE* = 125 ms), *t*(109) = 4.58, *p* < .001, *d*_z_ = 0.44, which is in line with previous studies (Bonin et al., 2014; Gelin et al., 2018; Mieth et al., 2019).

#### Recollection, familiarity, guessing, and detection of new words

To facilitate comparisons with prior research, the mean proportions of the different types of judgments by word type and animacy status are provided in Table 2. However, note that the hypotheses that are tested in the present experiment do not directly refer to these proportions but instead refer to the latent cognitive states into which these raw performance measures are decomposed as specified in the model-based analysis reported below.

**Table 2:** Mean proportions of “detailed recollection,” “feeling of familiarity,” “guessing,” and “new” judgments by word type and animacy status in Experiment 1

The old and new words’ animacy status	Judgment							
	“Detailed recollection”		“Feeling of familiarity”		“Guessing”		“New”	
Old animate	.53	(.02)	.23	(.01)	.06	(.01)	.19	(.01)
Old inanimate	.48	(.02)	.25	(.01)	.07	(.01)	.20	(.01)
New animate	.02	(< .01)	.07	(.01)	.05	(.01)	.86	(.01)
New inanimate	.03	(< .01)	.09	(.01)	.06	(.01)	.82	(.01)

Values in parentheses represent standard errors

**Fig. 1:**
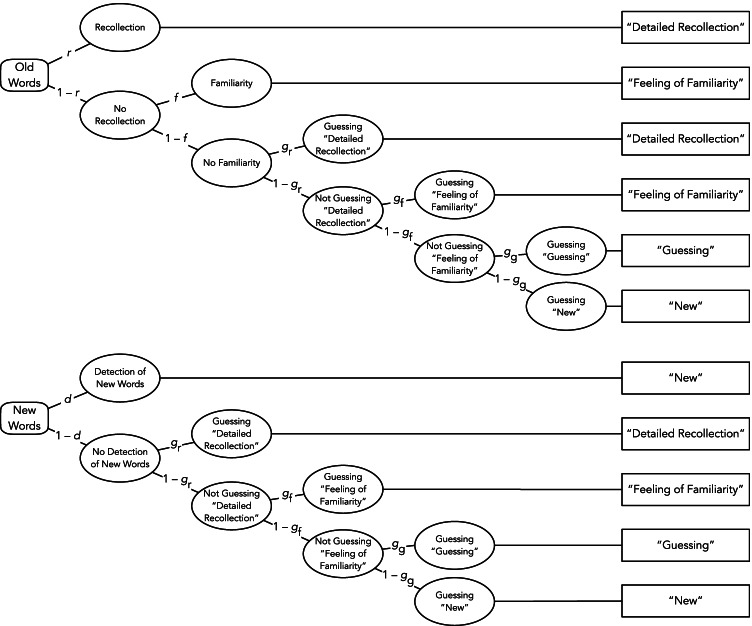
The four-states model by Erdfelder et al. (2007), adapted to the present experiment. Rounded rectangles on the left represent the words presented in the recognition test (old or new with respect to the encoding phase). The parameters attached to the branches of the trees denote transition probabilities between sequences of latent cognitive states (*r*: probability of recollection; *f*: conditional probability of familiarity in case of recollection failure; *g*_r_: conditional probability to guess “detailed recollection” in an uncertainty state; *g*_f_: probability to guess “feeling of familiarity” in an uncertainty state, conditional on not having guessed “detailed recollection”; *g*_g_: probability to choose “guessing” in an uncertainty state, conditional on not having guessed “detailed recollection” or “feeling of familiarity”; *d*: probability of detecting new words as new). The rectangles on the right represent the categories of observable responses

In Experiment 1, the results were analyzed with the multinomial four-states model by Erdfelder et al. (2007), shown in Fig. 1, to clearly distinguish between recollection, familiarity, guessing, and the detection of new words. Multinomial processing-tree models, to which class the applied model belongs, are stochastic models that explain observable response frequencies as a function of the postulated latent cognitive states or processes (for reviews, see Batchelder & Riefer, 1999; Erdfelder et al., 2009). The model’s parameters reflect these processes and are represented as probabilities varying between 0 and 1. Importantly, the model’s parameters for recollection and familiarity can be interpreted as reflecting only the processes they were intended to measure, since they are uncontaminated by the guessing processes that are represented by separate parameters for guessing “detailed recollection” and “feeling of familiarity” (Erdfelder et al., 2007).

To illustrate, the upper tree of Fig. 1 refers to the processing of old words presented during the encoding phase. An old word is recollected with probability *r*, resulting in a “detailed recollection” judgment. If participants do not have a detailed recollection of the word, which occurs with probability 1 – *r*, it may still appear familiar with the conditional probability *f*, triggering a “feeling of familiarity” judgment.^2^ If an old word is neither recollected nor familiar, which occurs with probability (1 – *r*) · (1 – *f*), guessing processes lead to a “detailed recollection” judgment with the conditional probability *g*_r_, to a “feeling of familiarity” judgment with the conditional probability (1 – *g*_r_) · *g*_f_, or to a “guessing” judgment with the conditional probability (1 – *g*_r_) · (1 – *g*_f_) · *g*_g_. Alternatively, participants may guess that the word was “new” with the conditional probability 1 – *g*_g_.

The lower tree of Fig. 1 refers to new words not presented in the encoding phase. New words can be correctly detected as new and thus be rejected with probability *d*. Detection fails with probability 1 – *d*, in which case guessing processes occur in the same way as for old words and result in “detailed recollection,” “feeling of familiarity,” “guessing,” or “new” judgments with the conditional probabilities *g*_r_, *g*_f_, *g*_g_, or 1 – *g*_g_, respectively.

To examine how animacy affects the parameters of the four-states model, two sets of the processing trees displayed in Fig. 1 were needed, one for animate and one for inanimate words. Parameter estimates and goodness-of-fit tests were calculated using multiTree (Moshagen, 2010). The four-states model is saturated (Erdfelder et al., 2007).

One advantage of multinomial processing-tree models is that they allow testing hypotheses directly at the level of the postulated processes. To illustrate, it is possible to formulate the to-be-tested hypothesis that recollection, reflected in parameter *r*, should differ between animate and inanimate words. This hypothesis can be implemented as an equality restriction by setting parameter *r* to be equal between animate and inanimate words. If the model including this equality restriction provides a significantly worse fit to the data than the base model not including this equality restriction, then it is necessary to conclude that recollection differs between animate and inanimate words.

We started the analysis by examining whether guessing differed between animate and inanimate words. The assumption that, in a state of uncertainty, guessing “detailed recollection” (*g*_r_), guessing “feeling of familiarity” (*g*_f_), and guessing “guessing” (*g*_g_) each do not differ between animate and inanimate words was compatible with the data; the model incorporating these restrictions fit the data, *G*^2^(3) = 3.90, *p* = .272, and was used as the base model for the following comparison of the recollection and the familiarity parameters between animate and inanimate words.

**Fig. 2:**
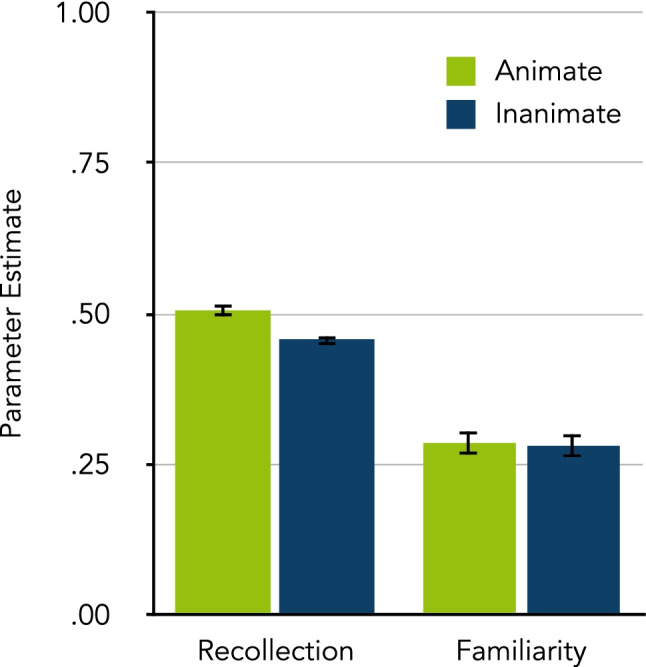
The quality of memory retrieval experiences according to the four-states model by Erdfelder et al. (2007), as estimated in Experiment 1. The parameter estimates of recollection (*r*) and familiarity-based (*f*) processes are shown separately for animate and inanimate words. The error bars represent standard errors

Figure 2 shows the estimates of the parameters representing recollection (*r*) and familiarity-based (*f*) processes for animate and inanimate words. Animate words were more likely to be recollected than inanimate words, Δ*G*^2^(1) = 19.68, *p* < .001, *w* = 0.03. Familiarity, by contrast, did not differ between animate and inanimate words, Δ*G*^2^(1) = 0.02, *p* = .877, *w* < 0.01.

However, the animacy advantage was not restricted to the recollection of words that had been present in the encoding phase. New animate words were also more likely to be detected as new than new inanimate words, Δ*G*^2^(1) = 26.06, *p* < .001, *w* = 0.04. Parameter *d* was significantly higher for animate (.71, *SE* = .01) than for inanimate words (.62, *SE* = .02). The estimates of the guessing parameters are reported in Table 3.

**Table 3:** Estimates of the guessing parameters in Experiments 1 and 2

The words’ animacy status	Experiment 1				Experiment 2	
	*g*_r_	*g*_f_	*g*_g_		*g*_i_	*g*_e_
Animate	.07 (.01)	.26 (.01)	.24 (.01)		.32 (.01)	.26 (.01)
Inanimate						

Experiment 1: Estimates of the guessing parameters of the four-states model by Erdfelder et al. (2007). The guessing parameters *g*_r_, *g*_f_, and *g*_g_ (see text for details) were each set to be equal between animate and inanimate words. Experiment 2: Parameters of guessing “old” in the inclusion test (*g*_i_) and in the exclusion test (*g*_e_) of the two-high threshold variant of the multinomial process-dissociation model by Buchner et al. (1995) were each set to be equal between animate and inanimate words within the base model. Values in parentheses represent standard errors

### Discussion

The results of Experiment 1 confirm the hypothesis derived from the richness-of-encoding account (Meinhardt et al., 2020) and the attentional account (Bugaiska et al., 2019) that animacy selectively enhances recollection. Even though animate words were encoded for a shorter period of time compared to inanimate words, animate words were more likely to be recollected than inanimate words (for a discussion of the relationship between response times and the richness of a word’s semantic representation, see Bonin et al., 2019). The animacy effect on the detection of new words was parallel to the animacy effect on the recollection of old words, as it should be (Glanzer & Adams, 1985). The parameters reflecting familiarity, by contrast, remained unaffected by animacy. Unlike Leding (2020), who found that animacy increased false-alarm rates, we did not find animacy effects on any of the three guessing parameters, allowing us to equate each of them between animate and inanimate words in our base model without significantly reducing the model fit. The present model-based analyses thus confirmed previous evidence (Bonin et al., 2014; Bugaiska et al., 2016; Rawlinson & Kelley, 2021) of animacy improving recognition memory by enhancing recollection but not familiarity.

Although we took guessing into account by applying the four-states model (Erdfelder et al., 2007) to the data of the remember-know-guess paradigm, the most important limitation of Experiment 1 is that the conclusions rest on self-reports of the participants’ experiential retrieval states. The results thus depend on the participants’ interpretation of what the response labels “detailed recollection” and “feeling of familiarity” were supposed to convey. To what extent, for example, the familiarity-based memory process as captured by the label “feeling of familiarity” corresponds to the process captured by the label “know” is open to debate (e.g., Pereverseff & Bodner, 2020; Williams & Lindsay, 2019; Williams & Moulin, 2015). Moreover, subjective experiences of recollection and familiarity may not invariably reflect objective memory accuracy. Research on memory of emotional events (Sharot et al., 2004; Talarico & Rubin, 2003) suggests that a subjectively intensified experience of recollection, which may result from increased arousal, does not always imply increased accuracy of memory. The natural vividness of animate beings may likewise make participants believe they are remembering vivid details when in fact they misinterpret these subjective feelings of vividness as enhanced recollection, yet without memory accuracy actually being enhanced. Our results do not support such an interpretation, since the four-states model (Erdfelder et al., 2007) was designed to provide process-pure measures of recollection and familiarity, and animacy was not associated with an increased tendency to guess “detailed recollection.” However, to arrive at clear conclusions about the link between animacy and recollection, it does not seem optimal to rely solely on measures that depend on the participants’ subjective interpretation of their experiential retrieval states.

Hence, Experiment 2 served to test whether the evidence for the enhanced recollection of animate words could be conceptually replicated using a procedure that allows for a performance-based measurement of the quality of recognition: the process-dissociation procedure (Jacoby, 1991). Similar to the remember-know(-guess) paradigm, the process-dissociation procedure serves to dissociate recollection from familiarity-based processes. However, rather than relying on subjectively experienced retrieval states and associated metacognitive beliefs about remembering, estimates of recollection and familiarity are derived from the participants’ objective capacity to follow the instructions in the memory test. Specifically, participants are confronted with three types of words in a recognition test: Critical words learned in a first encoding phase (Phase 1), words learned in a second encoding phase (Phase 2), and new words not presented during encoding (cf. Buchner et al., 1995). Two types of test instructions are given: In the *inclusion test*, participants have to respond “old” to all words presented during encoding and “new” to words that were not seen during encoding. In the *exclusion test*, the “old” response is reserved for words from Phase 2. Participants have to reject (or exclude) all critical words from Phase 1 and call them “new,” just like the words not encountered before. Once guessing is taken into account, correct “old” responses to Phase-1 words are assumed to arise from both recollection and familiarity in the inclusion test. In the exclusion test, by contrast, recollection facilitates but familiarity impedes avoiding false “old” responses to Phase-1 words. From these assumptions, measures of recollection and familiarity are derived (Jacoby, 1991). Parallel to the methodological approach taken in Experiment 1, we used multinomial modeling to disentangle recollection, familiarity, and guessing processes by applying a variant of the well-validated multinomial process-dissociation model (Buchner et al., 1995). The predictions for the recollection and familiarity parameters are identical to those for the subjective measures in Experiment 1: Animacy should selectively enhance recollection but not familiarity.

## Experiment 2

### Method

#### Participants

Experiment 2 was made available online using SoSci Survey (Leiner, 2020). Participation was only possible with a desktop or laptop computer. The experiment was advertised on social media and via email. All participants but one were students. The final sample contained data from 163 participants (125 female) with a mean age of 24 years (*SD* = 7). They were randomly assigned to either the inclusion test (*n* = 81) or the exclusion test (*n* = 82). Eighty-three additional data sets could not be included in the analysis because the participants did not complete the experiment (30 participants dropped out even before they had given informed consent, presumably following our instructions to close the browser window if they were not in a distraction-free environment; 18 dropped out when they were asked about their demographic data; 35 participants – 18 assigned to the inclusion and 17 to the exclusion test – dropped out after they had started the encoding task). We used all but four of the words used in Experiment 1 to achieve comparability between the experiments. Given that in the process-dissociation procedure the analysis is based only on Phase-1 words and new words (while Phase-2 words are typically not included in the analysis; see Buchner et al., 1995; Jacoby, 1991), it was necessary to use a slightly larger sample of *N* = 163 participants to compensate for the lower number of data points in the recognition test to achieve the same level of sensitivity of the model-based statistical analysis. A sensitivity analysis indicated that with this sample size, 104 relevant data points in the recognition test, and α = .05, an effect of animacy on the multinomial process-dissociation model’s recollection and familiarity parameters of the size *w* = 0.03 could be detected with a statistical power of 1 – β = .95 (Faul et al., 2007). Participation was compensated by course credit or the chance to win a € 20 online voucher. All participants gave written informed consent prior to participation. Approval was obtained from the ethics committee of the Faculty of Mathematics and Natural Sciences at Heinrich Heine University Düsseldorf for a series of animacy experiments to which the present experiment belongs. Minor adjustments were necessary due to the COVID-19 pandemic that did not require additional ethics approval. The experiment was conducted in accordance with the Declaration of Helsinki.

#### Materials

The stimulus set was created by excluding two animate and two inanimate words from the stimulus set used in Experiment 1 to ensure that three equally sized, non-overlapping stimulus subsets could be created. These subsets consisted of 26 animate and 26 inanimate words each, were randomly selected without replacement for each participant from the lists of 78 animate and 78 inanimate words, and were presented in two encoding phases and as new words in the test phase. The animate and inanimate word lists were matched on ten mnemonically relevant dimensions (see Table 4).

**Table 4:** Dimensions on which animate and inanimate word lists were matched in Experiment 2

Dimension (range of the rating scale)	Animate		Inanimate		Comparison
	*M*	*SD*	*M*	*SD*	
Semantic typicality (1–7)	2.39	0.94	2.61	1.36	*t*(137.17) = 1.21, *p* = .228, *d* = 0.19
Age of acquisition (1–7)	3.35	1.20	3.42	1.18	*t*(154) = 0.36, *p* = .718, *d* = 0.06
Conceptual familiarity (1–5)	3.19	0.58	3.33	0.82	*t*(138.11) = 1.29, *p* = .199, *d* = 0.21
Word frequency	9.33	17.82	8.89	18.46	*t*(154) = 0.15 *p* = .878, *d* = 0.02
Number of phonemes	5.03	1.55	4.92	1.37	*t*(154) = 0.44, *p* = .661, *d* = 0.07
Number of syllables	2.03	0.81	2.00	0.66	*t*(148.64) = 0.22, *p* = .829, *d* = 0.03
Number of letters	6.14	1.63	5.77	1.53	*t*(154) = 1.47, *p* = .144, *d* = 0.24
Concreteness (1–7)	5.17	0.48	5.09	0.49	*t*(154) = 1.07, *p* = .285, *d* = 0.17
Meaningfulness (1–7)	3.64	0.73	3.59	0.76	*t*(154) = 0.47, *p* = .637, *d* = 0.08
Imagery (1–7)	5.48	0.94	5.48	0.96	*t*(154) < 0.01, *p* = .998, *d* < 0.01

In Experiment 2, two animate and two inanimate words were excluded from the stimulus set used in Experiment 1. Norming data were taken from Schröder et al. (2012) and Meinhardt et al. (2020). Words were selected so as to minimize, for each dimension, the mean differences between the lists of animate and inanimate words. The rightmost column shows that the lists of animate and inanimate words did not differ significantly on any of the controlled dimensions

#### Procedure

In Phase 1, the critical encoding phase, a mixed list of 26 animate and 26 inanimate words was presented in random order. As in Experiment 1, participants incidentally learned the words while performing a self-paced animacy-rating task. In Phase 2, participants were instructed to intentionally learn a new mixed list of 26 animate and 26 inanimate words. As in Phase 1, Phase-2 words were shown in random order at the center of the browser window in 20-point Arial font. In each trial of the intentional learning task, participants saw the word for 1 s before a “next” button appeared. The word was displayed until the participant pressed the button to proceed. To facilitate discrimination between Phase-1 words and Phase-2 words, Phase-1 words were shown in blue font color and Phase-2 words in red font color.

The recognition test was separated from Phase 2 by a short distractor task with a mean duration of 37 s (*SD* = 13), in which participants completed ten simple mathematical equations (*M* = 98 % correct). In the test phase, the 104 words from both encoding phases were randomly intermixed with 26 new animate words and 26 new inanimate words, resulting in 156 words being presented in black font color. Depending on the test condition, participants completed one of two different recognition tests conforming to Jacoby’s (1991) process-dissociation procedure. Inclusion versus exclusion tests were manipulated between subjects because the multinomial process-dissociation model had been validated using between-subjects designs (Buchner et al., 1995), and we did not want to confuse participants with varying test instructions. Participants performing the inclusion test were instructed to choose “old” for the blue words rated in Phase 1 *and* for the red words intentionally learned in Phase 2. Words they had not seen before had to be judged “new.” Participants performing the exclusion test were instructed to select “old” *only* for the red words they had intentionally learned in Phase 2. Blue words from Phase 1 had to be rejected and called “new,” just like the words they had not seen before. Participants were instructed that if a word had been presented in blue font color in the rating task, the same word could not have been among the words that had to be retained for the recognition test. In each trial of the recognition test, a word (presented at the center of the browser window) had to be classified as “old” or “new.” At the bottom of the browser window, condition-specific instructions remained visible throughout the test. Once the word was classified as “old” or “new,” participants clicked a “next” button to proceed to the next word.

After the recognition test, participants were asked to report whether they remembered the instructions, whether they followed the instructions, and whether all stimuli were accurately presented.^3^ Thereafter, participants were compensated, debriefed, and thanked for their time. The experiment lasted roughly 30 min.

### Results

#### Ratings and response times

As expected, animacy ratings were higher for animate words (*M* = 6.34, *SE* = 0.07) than for inanimate words (*M* = 1.96, *SE* = 0.08), *t*(162) = 30.37, *p* < .001, *d*_z_ = 2.38. After applying an outlier correction (excluding response times that deviated by ± 3 *SD* from the individual mean), it was again found that participants rated animate words (*M* = 3,324 ms, *SE* = 97 ms) faster than inanimate words (*M* = 3,572 ms, *SE* = 107 ms), *t*(162) = 3.96, *p* < .001, *d*_z_ = 0.31.

#### Recollection, familiarity, and guessing

To facilitate comparisons with prior research, the mean proportions of “old” and “new” judgments are presented as a function of word type, animacy status, and test condition in Table 5. However, note that our hypotheses do not directly refer to these proportions but instead refer to the latent cognitive states into which these raw performance measures are decomposed as specified in the model-based analysis reported below.

**Table 5:** Mean proportions of “old” and “new” judgments for old words presented in Phase 1 and new words as a function of animacy status and test condition in Experiment 2

The old and new words’ animacy status	Inclusion test				Exclusion test			
	Judgment				Judgment			
	“Old”		“New”		“Old”		“New”	
Old animate	.84	(.01)	.16	(.01)	.28	(.02)	.72	(.02)
Old inanimate	.80	(.02)	.20	(.02)	.30	(.02)	.70	(.02)
New animate	.15	(.01)	.85	(.01)	.12	(.02)	.88	(.02)
New inanimate	.16	(.01)	.84	(.01)	.13	(.02)	.87	(.02)

Values in parentheses represent standard errors

**Fig. 3:**
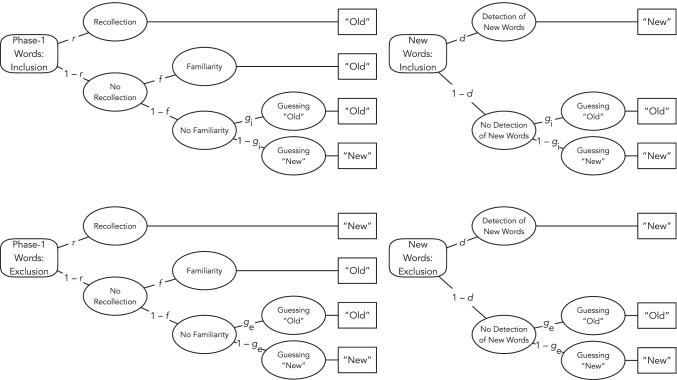
The multinomial process-dissociation model by Buchner et al. (1995), adapted to the present experiment. Rounded rectangles on the left represent the presented words (old Phase-1 words or new words) of the recognition test. The upper trees refer to words presented in the inclusion test and the lower trees to words presented in the exclusion test. The parameters attached to the branches of the trees denote transition probabilities between sequences of latent cognitive states (*r*: probability of recollection; *f*: conditional probability of familiarity in case of recollection failure; *g*_i_: conditional probability to guess “old” in an uncertainty state in the inclusion test; *g*_e_: conditional probability to guess “old” in an uncertainty state in the exclusion test; *d*: probability of detecting new words as new). The rectangles on the right represent the categories of observable responses

To disentangle recollection, familiarity, and guessing processes, we used a variant of the well-established multinomial process-dissociation model proposed and validated by Buchner et al. (1995) as presented in Fig. 3. Following the process-dissociation procedure (Buchner et al., 1995; Jacoby, 1991), only the cognitive processes that occur in response to Phase-1 words and new words are displayed. The sequences of processes that lead to “old” or “new” judgments in the inclusion and exclusion tests are illustrated in the upper and lower trees, respectively.

A Phase-1 word in the inclusion test (upper left tree in Fig. 3) is assumed to be recollected with probability *r*, resulting in an “old” judgment. If participants do not recollect a Phase-1 word, which occurs with probability 1 – *r*, the word may still appear familiar with the conditional probability *f*, thus prompting an “old” judgment.^4^ If a word is neither recollected nor familiar, which occurs with probability (1 – *r*) · (1 – *f*), the word is guessed to be “old” with the conditional probability *g*_i_ or guessed to be “new” with probability 1 – *g*_i_.

In the exclusion test (lower left tree in Fig. 3), a Phase-1 word is assumed to be recollected with probability *r*. Exclusion instructions require participants to reject Phase-1 words. Therefore, recollection leads to a “new” judgment. If participants fail to recollect a Phase-1 word, which occurs with probability 1 – *r*, it may still appear familiar with the conditional probability *f*, triggering an “old” judgment. If a word is neither recollected nor familiar, which occurs with probability (1 – *r*) · (1 – *f*), the word is guessed to be “old” with the conditional probability *g*_e_ or guessed to be “new” with probability 1 – *g*_e_.

The model depicted in Fig. 3 deviates from the original model by Buchner et al. (1995) in that it incorporates the assumption that a new word can be detected as new with probability *d*, prompting a “new” judgment (trees on the right side of Fig. 3). It has been shown that the model that includes this assumption provides an even better fit to the validation data by Buchner et al. than a model that denies that new words can be detected (Erdfelder & Buchner, 1995), consistent with the general pattern that two-high threshold models (including the detection of new words) perform better than one-high threshold models (denying the detection of new words) in validation studies (Bayen et al., 1996; Snodgrass & Corwin, 1988). If detection fails, which occurs with probability 1 – *d*, guessing processes result in an “old” judgment with the conditional probabilities *g*_i_ and *g*_e_ in the inclusion test and the exclusion test, respectively. Alternatively, a word is guessed to be “new” with probabilities 1 – *g*_i_ in the inclusion test and 1 – *g*_e_ in the exclusion test.

To test how animacy affects the memory and guessing parameters, two sets of the processing trees displayed in Fig. 3 were needed, one for animate and one for inanimate words. Without imposing additional equality restrictions, the model is not identifiable. As suggested by Erdfelder and Buchner (1995), we adopted the assumption that the probability of recollecting an old word (*r*) is equal to the probability of detecting a new word as new (*d*), separately for animate and inanimate words, since Erdfelder and Buchner showed that the model including this restriction performed better in validation tests than alternative models.^5^ This restriction resulted in a saturated model. Parallel to Experiment 1, we started our analysis by examining whether guessing differed between animate and inanimate words. The assumptions that guessing “old” in the inclusion test (*g*_i_) does not differ between animate and inanimate words and that guessing “old” in the exclusion test (*g*_e_) does not differ between animate and inanimate words were compatible with the data; the model incorporating these restrictions fit the data, *G*^2^(2) = 0.02, *p* = .990, and was used as the base model to estimate the parameters and to test our hypotheses.

**Fig. 4:**
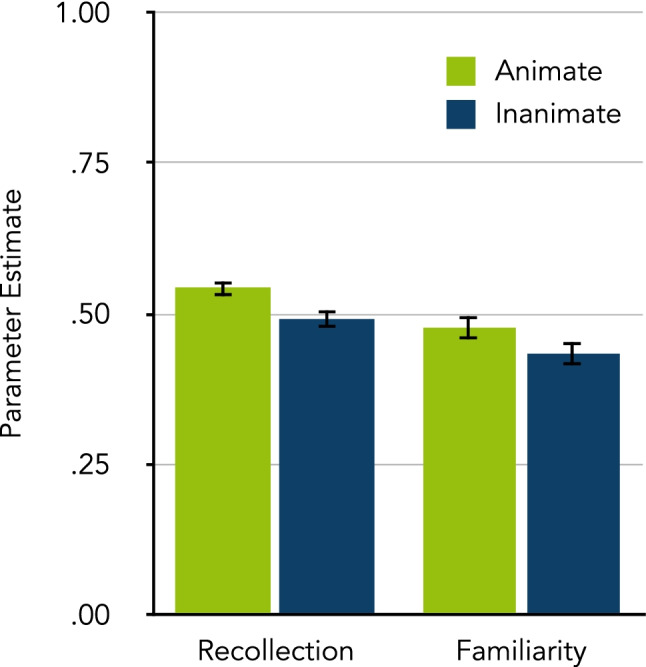
The quality of recognition according to the two-high threshold variant of the multinomial process-dissociation model by Buchner et al. (1995), as estimated in Experiment 2. The parameter estimates of recollection (*r*) and familiarity-based (*f*) processes are shown separately for animate and inanimate words. The error bars represent standard errors

Figure 4 shows the parameter estimates of recollection (*r*) and familiarity-based (*f*) processes for animate and inanimate words. The probability of recollecting animate words was higher than that of inanimate words, Δ*G*^2^(1) = 11.36, *p* < .001, *w* = 0.03. By contrast, animate and inanimate words did not differ in familiarity, Δ*G*^2^(1) = 2.64, *p* = .104, *w =* 0.01, although, descriptively, animate words were more likely to appear familiar than inanimate words. The estimates of the guessing parameters are reported in Table 3.

### Discussion

The results of Experiment 2 confirm those of Experiment 1. Crucially, animate words were more likely to be recollected than inanimate words. Animacy did not significantly enhance familiarity-based processing, although animate compared to inanimate words were descriptively more likely to be judged based on familiarity. Again, guessing did not differ between animate and inanimate words. We thus conclude that animacy is associated with enhanced recollection. This conclusion is in line with the results of previous studies examining related constructs such as source memory (Gelin et al., 2018; Mieth et al., 2019).

## General discussion

While the effect of animacy on free recall turned out to be a robust phenomenon (e.g., Bonin et al., 2014; Meinhardt et al., 2020; Nairne et al., 2013; Popp & Serra, 2016), data from recognition paradigms have provided only mixed support for an animacy advantage (e.g., Bonin et al., 2014; Leding, 2020; Mieth et al., 2019). Specifically, Leding (2020) reported that participants recognized more animate than inanimate words but that they also committed more false alarms on animate than on inanimate words, such that the recognition benefits were completely eliminated once guessing was taken into account. Based on these findings in isolation, it seemed possible to postulate that animacy primarily affects guessing but not memory accuracy. However, this *guessing hypothesis* contrasts with findings in the remember-know(-guess) paradigm, suggesting that animacy enhances correct recognition without affecting false-alarm rates (Bonin et al., 2014; Bugaiska et al., 2016; Rawlinson & Kelley, 2021). A possible reason for the discrepancy across studies is that animacy may not equally enhance all processes underlying recognition memory performance. Proximate mechanisms of the animacy effect such as the richness-of-encoding account (Meinhardt et al., 2020) and the attentional account (Bugaiska et al., 2019) imply the hypothesis that animacy selectively enhances recollection but not familiarity. This *recollection hypothesis* was tested in the present experiments. Both experiments provide a convergent pattern of results: Animacy enhances recollection in recognition paradigms. By contrast, familiarity and guessing are not significantly affected.

We started by examining subjectively experienced retrieval states in the remember-know-guess paradigm (Gardiner et al., 1996; Gardiner et al., 1997). Essentially, the subjective experience and associated metacognitive beliefs about remembering are supposed to be different for words that are recollected and for words that appear familiar. When examining the subjective experience of recollection and familiarity, it is important to consider guessing given that, a priori, it seemed possible that the vividness of animate beings may induce participants to guess that an animate word was recollected. Instead of comparing the raw frequencies of “remember,” “know,” and “guess” judgments on recognition hits and false alarms of animate and inanimate words, as was done in previous studies (Bonin et al., 2014; Bugaiska et al., 2016; Rawlinson & Kelley, 2021), we used the well-validated four-states model of memory retrieval experiences (Erdfelder et al., 2007) to obtain process-pure measures of recollection and familiarity. In line with the recollection hypothesis, animacy was selectively associated with enhanced recollection without familiarity being affected. Corresponding to the recollection advantage for old animate words, new animate words were also more likely to be detected as new than new inanimate words (see Glanzer & Adams, 1985). These findings thus suggest that the recognition advantage of animate beings is primarily driven by enhanced recollection. At the same time, we need to reject the guessing hypothesis given that the assumption that guessing does not differ between animate and inanimate words was compatible with the data.

While Experiment 1 provides further support for the recollection hypothesis of the animacy advantage, it may be problematic to solely focus on subjectively experienced recollection states to test whether animate words are better recollected than inanimate words. With the remember-know-guess paradigm, Experiment 1 necessarily relies on the participants’ introspective ability to judge the quality of their retrieval states. However, it has been observed that subjective and objective measures of memory may diverge. For instance, it has been shown that emotional events may provoke flashbulb memories that are experienced as extremely vivid. This vividness, however, might reflect the arousal associated with the emotional event rather than the quality of remembering (Sharot et al., 2004; Talarico & Rubin, 2003). Accordingly, one could speculate that animate beings might elicit more vivid imagery than inanimate objects, which may boost recollection judgments. It thus should not be taken for granted that subjective and objective aspects of remembering converge without explicitly testing this assumption.

We therefore sought to conceptually replicate the effect of animacy on recollection by using a paradigm that relies on objective memory performance. In Experiment 2, participants were divided into two groups and were instructed to either include or exclude words that were previously presented in the first of two encoding phases according to the process-dissociation procedure (Jacoby, 1991). Recollection is reflected in superior performance in inclusion and exclusion tests. While both recollection and familiarity increase the probability of recognizing a word as “old” in the inclusion test, these two processes oppose each other in the exclusion test: Recollection allows participants to reject Phase-1 words, whereas familiarity prompts them to accept those words. In order to take guessing into account, parameters representing the probability of recollection and familiarity-based processes were estimated using a variant of the well-validated multinomial process-dissociation model (Buchner et al., 1995). The results suggest that, with respect to the animacy effect, the conclusions that can be drawn from subjective and objective measures of recollection and familiarity converge. Experiment 2 conceptually replicated the results of Experiment 1: Animacy was associated with enhanced recollection but not familiarity, confirming the recollection hypothesis. Furthermore, the data again forced us to reject the guessing hypothesis. The words’ animacy status did not affect the guessing parameters. The present data thus confirm previous studies using the remember-know(-guess) paradigm (Bonin et al., 2014; Bugaiska et al., 2016; Rawlinson & Kelley, 2021) and extend these studies by showing that subjective and objective measures of recollection converge. Just like any measurement method, both the remember-know-guess paradigm (e.g., Umanath & Coane, 2020; Williams & Lindsay, 2019) and the process-dissociation procedure (e.g., Dodson & Johnson, 1996; Jacoby, 1998; Joordens & Merikle, 1993; for a review, see Yonelinas & Jacoby, 2012) have their limitations. The strength of the evidence presented here lies in demonstrating that methods as different as these two led to the same result with regard to the animacy effect on memory.

Due to the evolutionary-adaptive significance of living beings such as predators, prey, and mating partners, one could argue that, from an ultimate perspective, it might be adaptive that animacy enhances recollection but not familiarity. For instance, survival chances might increase if the memory trace of prey comprises a detailed recollection of the context of a previous encounter (so one knows where and when to go for hunting) rather than an acontextual memorial experience of having met a specific type of animal before. Regardless of the adaptive value of the converging evidence on how animacy affects the quality of remembering, the present findings provide insights into the proximate mechanisms underlying the animacy effect. In general, mechanistic explanations should account for the fact that animacy affects not only the quantity of remembering but also its quality. A promising explanation of the animacy effect refers to the notion that animate words are associated with a richer encoding than inanimate words (Meinhardt et al., 2020; Rawlinson & Kelley, 2021). Meinhardt et al. (2020) found that their participants spontaneously generated more ideas in response to animate than inanimate words (see also Bonin et al., 2022). Some of these associations may still be available at test and serve as effective retrieval cues in free-recall paradigms. The richness-of-encoding account thus implies that in recognition paradigms, participants should not only recognize the word but also have access to the associatively rich processing of the word that occurred during encoding, which is supposed to be experienced as enhanced recollection in the recognition test.

Another potentially related explanation of the animacy effect refers to the notion that animate words are more potent than inanimate words in capturing attention during encoding. This attentional account (Bugaiska et al., 2019) seems plausible, since animate beings and animate properties (such as animate movements) are often prioritized in perception and attention (New et al., 2007). As recollection is often assumed to be a resource-dependent process (Gardiner et al., 1996; Yonelinas, 2002), the attentional account fits the present data well. The richness-of-encoding account and the attentional account are regarded as complementary rather than competitive, since increased attention during encoding may eventually lead to richer representations (Meinhardt et al., 2020; Mieth et al., 2019). However, it seems important to mention that the more detailed recollection of animate words at retrieval is still better supported by the available data than the dependence of the animacy effect on attentional resources at encoding, for which inconsistent results have been reported (Bonin et al., 2015; Bugaiska et al., 2019; Leding, 2019; Rawlinson & Kelley, 2021). Whether the controlled allocation of attentional resources is necessary to generate and activate rich semantic representations of animate words is an open issue (Bonin et al., 2015; Rawlinson & Kelley, 2021). In the future, it will be important to disentangle these two accounts. One possibility would be to try to manipulate the postulated underlying processes – that is, attention and richness of encoding – more directly. For instance, to test the richness-of-encoding account of the animacy effect, one may rely on manipulations that have proven useful to test richness of encoding as an explanation of the survival-processing effect (Kroneisen & Erdfelder, 2011).

In summary, the present results once more confirm the hypothesis derived from the adaptive-memory framework proposed by Nairne and co-workers (Nairne et al., 2013; Nairne & Pandeirada, 2016) that animacy is associated with a memory advantage. Originally, the animacy effect refers to the quantity of remembering only: More animate than inanimate words are typically recalled in free-recall paradigms and other memory tests (Nairne et al., 2013; Nairne et al., 2017). The present results suggest that animacy affects not only the quantity of remembered information but also the subjectively experienced and objectively measurable qualitative aspects of remembering. Not only did we model the quality of recognition at the process level, but we also examined the animacy effect in two fundamentally different experimental procedures. This allowed us to tap into complementary methods to assess the quality of remembering, both subjectively with the remember-know-guess paradigm and objectively by means of the process-dissociation procedure, in order to comprehensively understand how animacy improves recognition. The results suggest that, with respect to the animacy effect, objective and subjective aspects of remembering converge: Both experiments consistently showed that animacy enhances recollection but affects neither familiarity nor guessing. Mechanistic explanations of the animacy effect thus have to account for both quantitative and qualitative changes in remembering. In this sense, the present results not only help to understand the animacy effect itself but might also promote refining the functional understanding of memory and its mechanistic underpinnings in general.

## Footnotes

1 Based on their responses, it would have been possible to exclude the data of two more participants. However, this would not have changed any statistical conclusions, so we decided to include these data sets into the final analysis, following a recommendation of Elliott et al. (2022).

2 Note that parameter *f* and the corresponding guessing parameter *g*_f_ refer to the parameters *k* and *g*_k_ of the original model by Erdfelder et al. (2007). The parameter labels were adapted to the labels of the recollection and familiarity judgments used in the present experiment, which also allows us to maintain consistency when referring to the recollection and familiarity-based processes across our two experiments.

3 Based on their responses, it would have been possible to additionally exclude data from 23 participants. However, this would not have changed any statistical conclusions, so we decided to include these data sets into the final analysis, following a recommendation of Elliott et al. (2022).

4 Note that the parameters *r* and *f* correspond to the parameters *c* and *u*_c–_ (referring to “conscious” and “unconscious” memory), respectively, of the extended measurement model for the process-dissociation procedure proposed by Buchner et al. (1995). The parameter labels were adapted to maintain consistency when referring to the recollection and familiarity-based processes across experiments and to use labels that are more neutral in terms of the subjective experience of the underlying processes (e.g., familiarity in the process-dissociation procedure does not necessarily imply that the process is completely unconscious).

5 While the two-high threshold model does not only provide a better fit to the validation data (Erdfelder & Buchner, 1995) but also to the present data than the one-high threshold model originally proposed by Buchner et al. (1995), the assumption that the recollection of old words is equal to the detection of new words is not critical to the results reported here, since the conclusions about the animacy effects on recollection, familiarity, and guessing processes remain the same regardless of whether the two-high threshold or the original one-high threshold variant of the model is applied to the data.
